# Predicting vigilance vulnerability during 1 and 2 weeks of sleep restriction with baseline performance metrics

**DOI:** 10.1093/sleepadvances/zpac040

**Published:** 2022-10-25

**Authors:** June C Lo, Jit Wei A Ang, Tiffany B Koa, Ju Lynn Ong, Julian Lim

**Affiliations:** Centre for Sleep and Cognition, Yong Loo Lin School of Medicine, National University of Singapore, Singapore, Singapore and; Department of Psychology, National University of Singapore, Singapore, Singapore; Centre for Sleep and Cognition, Yong Loo Lin School of Medicine, National University of Singapore, Singapore, Singapore and; Centre for Sleep and Cognition, Yong Loo Lin School of Medicine, National University of Singapore, Singapore, Singapore and; Centre for Sleep and Cognition, Yong Loo Lin School of Medicine, National University of Singapore, Singapore, Singapore and; Centre for Sleep and Cognition, Yong Loo Lin School of Medicine, National University of Singapore, Singapore, Singapore and; Department of Psychology, National University of Singapore, Singapore, Singapore

**Keywords:** baseline performance, diffusion drift model, sleep restriction, sustained attention, vigilance, vulnerability

## Abstract

**Study Objectives:**

We attempted to predict vigilance performance in adolescents during partial sleep deprivation using task summary metrics and drift diffusion modelling measures (DDM) derived from baseline vigilance performance.

**Methods:**

In the Need for Sleep studies, 57 adolescents (age = 15–19 years) underwent two baseline nights of 9-h time-in-bed (TIB), followed by two cycles of weekday sleep-restricted nights (5-h or 6.5-h TIB) and weekend recovery nights (9-h TIB). Vigilance was assessed daily with the Psychomotor Vigilance Task (PVT), with the number of lapses (response times ≥ 500 ms) as the primary outcome measure. The two DDM predictors were drift rate, which quantifies the speed of information accumulation and determines how quickly an individual derives a decision response, and non-decision time range, which indicates within-subject variation in physical, non-cognitive responding, e.g. motor actions.

**Results:**

In the first week of sleep curtailment, faster accumulation of lapses was significantly associated with more lapses at baseline (*p* = .02), but not the two baseline DDM metrics: drift and non-decision time range (*p* > .07). On the other hand, faster accumulation of lapses and greater increment in reaction time variability from the first to the second week of sleep restriction were associated with lower drift (*p* < .007) at baseline.

**Conclusions:**

Among adolescents, baseline PVT lapses can predict inter-individual differences in vigilance vulnerability during 1 week of sleep restriction on weekdays, while drift more consistently predicts vulnerability during more weeks of sleep curtailment.

**Clinical Trial Information:**

Effects of Napping in Sleep-Restricted Adolescents, clinicaltrials.gov, NCT02838095. The Cognitive and Metabolic Effects of Sleep Restriction in Adolescents (NFS4), clinicaltrials.gov, NCT03333512.

**Statement of Significance**

In adults, the extent to which sleep deprivation impairs vigilance can be predicted using performance on vigilance tests in a well-rested state. Both summary metrics (e.g. lapses in attention), and metrics derived from computational models are useful in this regard. Here, we extend these findings by showing that the same is true of adolescents over multiple nights of sleep restriction, although the pattern of prediction in this analysis was more nuanced. This is the first study to demonstrate that baseline vigilance is a robust predictor in this earlier maturational stage. Knowing one’s vulnerability to sleep restriction may motivate behavioral change, and future studies could build on this model with the inclusion of other relevant chronobiological and physiological factors.

## Introduction

Recurrent partial sleep loss has clear adverse effects on many facets of cognition, with vigilance being particularly affected [1]. While this has been a relatively uncontroversial finding in adults [2], the consensus is only more recently emerging that the same is true for adolescents – contrasting with earlier studies suggesting that adolescents could be resilient to sleep loss [3]. For example, Short et al. [4] and Campbell et al. [5] both found dose-dependent responses of sleep duration on adolescents’ vigilance, which was measured with the Psychomotor Vigilance Test (PVT) [6], in titrated, laboratory paradigms. In the Need For Sleep (NFS)^1^ studies [7–11], which we reanalyzed in this communication, adolescents’ PVT performance, working memory, and processing speed were also consistently impaired in several different short sleep schedules relative to a 9-h time-in-bed (TIB) control group.

Beyond the population-level consequences of short sleep, a subset of individuals may be particularly vulnerable to sleep loss; this might be inferred from the large inter-individual variability in vigilance declines across single exposures to sleep curtailment [5, 7–14]. Although this has not been explicitly tested in adolescents, this vulnerability is stable across multiple exposures to sleep deprivation in adults [15, 16], suggesting that it may be useful to find its markers and predictors in younger people.

While in adults, baseline measures of sleep latency on the Maintenance of Wakefulness Test [17], as well as connectivity metrics derived from functional magnetic resonance imaging [18] may predict individual differences in vigilance declines during sleep loss, the most consistent predictor with acute total sleep deprivation or multi-night partial sleep loss is performance on identical tasks while well rested. Predictive power for impairment associated with total sleep deprivation has been demonstrated for both summary metrics (e.g. PVT lapses) [19] and measures derived from drift-diffusion modeling (DDM) [20].

The DDM, which was first introduced by Ratcliff [21], posits that every decision-making process consists of a non-decision-making phase and a decision-making phase. The former involves an element of physical, non-cognitive responding, such as motor actions, and can be indicated by non-decision time which includes the time it takes to initiate a physical response. The decision-making phase is the cognitive process of deriving a final decision, which can be objectively quantified as the drift rate—the speed of information accumulation that determines how quickly an individual derives a decision response. For each individual, an average non-decision time and drift measure and their corresponding variability measure (e.g. range and standard deviation) can be derived based on their performance across PVT trials. DDM metrics have been used as a predictor of behavioral performance in multiple studies involving the effects of sleep deprivation on standard PVT performance [20, 22], and these have consistently shown that lower drift rates at baseline are associated with poorer task performance during sleep deprivation. In addition, variability/range in non-decision time across all trials is greater after sleep deprivation than at baseline [20, 23] and among vulnerable as compared to resilient participants [22].

Currently, only one study has used baseline PVT metrics to predict inter-individual differences in vigilance vulnerability during sleep restriction across multiple nights on adults [17]; this report showed that the baseline number of lapses could predict its increase over 5 nights of 4-h time-in-bed (TIB), without exploring the predictive power of DDM measures.

Whether summary metrics (e.g. number of lapses) and DDM parameters from the PVT in well-rested adolescents similarly predict their vulnerability to multi-night sleep restriction remains to be investigated. Importantly, whether parameters predicting vulnerability to sleep restriction on weekdays over 1 week are the same as those predicting vigilance vulnerability when sleep restriction is recurrent and occurs again after the typical recovery sleep over the weekend is not yet known. To address these in the present report, we used PVT data collected from adolescents who went through two cycles of sleep restriction of 3–5 nights, separated by two nights of recovery sleep. We separately examined performance declines in the first week of sleep curtailment, and further declines from the first to the second week, which represents vigilance vulnerability to recurrent periods of sleep restriction. Based on the findings of Patanaik et al. [22]^.^ and Chua et al. [24], we predicted that both of these measures of vulnerability would be associated with more lapses and a lower drift rate at baseline.

## Methods

### Participants

Data from 57 adolescent participants (31 males) between 15 and 19 years of age in the NFS Studies [8, 9] were used in the present analysis (Table 1). During the screening, participants’ sleep was assessed with the Pittsburgh Sleep Quality Index (PSQI) [25], and actigraphy accompanied by a sleep diary. Their chronotype was measured with the Morningness–Eveningness Questionnaire [26], excessive daytime sleepiness with the Epworth Sleepiness Scale [27], and symptoms of chronic sleep reduction with the Chronic Sleep Reduction Questionnaire [28] (for details of screening, refer to [7]).

**Table 1.** Characteristics of the participants measured during the screening phase

	Overall		5 h group		6.5 h group		*P*
	Mean	SD	Mean	SD	Mean	SD	
*N*	57	—	28	—	29	—	—
Age (years)	16.74	1.13	16.91	1.14	16.58	1.12	.28
Gender (% male)	54.38	—	57.14	—	51.72	—	.68
Body Mass Index	21.09	3.12	20.92	2.77	21.25	3.46	.69
Daily caffeine intake (cups)	0.66	0.85	0.75	0.91	0.58	0.80	.46
Morningness-Eveningness Questionnaire	49.60	7.56	50.25	7.66	48.97	7.54	.53
Epworth Sleepiness Scale	7.40	3.24	6.57	2.86	8.21	3.43	.06
Chronic Sleep Reduction Questionnaire	34.73	5.52	34.21	5.07	35.24	5.96	.49
Pittsburgh Sleep Quality Index							
Weekday TIB (h)	6.69	1.09	6.52	0.72	6.85	1.35	.26
Weekend TIB (h)	8.85	1.13	8.76	1.09	8.93	1.18	.57
Weekday TST (h)	6.30	1.00	6.13	0.73	6.46	1.19	.21
Weekend TST (h)	8.48	1.11	8.40	1.02	8.56	1.20	.59
Global score	4.93	1.94	5.39	2.25	4.48	1.50	.08
Actigraphy							
Weekday TIB (h)	6.72	0.92	6.44	0.99	7.00	0.77	**.02**
Weekend TIB (h)	8.30	0.95	8.15	0.70	8.45	1.13	.24
Weekday TST (h)	5.60	0.82	5.69	0.89	5.51	0.75	.41
Weekend TST (h)	6.99	0.95	7.23	0.63	6.76	1.14	.06
Average TST (h)	6.00	0.67	6.14	0.64	5.86	0.68	.12
Sleep Efficiency (%)	83.68	6.82	88.51	4.10	79.02	5.57	**<.001**

Because of missing data, for actigraphy, *n* = 26–28 for the 5 h group.

Eligibility criteria included no known health conditions or sleep disorders, a body mass index of ≤30 kg/m^2^, a daily intake of ≤5 cups of caffeinated beverages, not habitual short sleepers (actigraphically estimated average TIB of <6 h with weekend sleep extension of ≤1 h), and no travel history across >2 time zones in the month prior to the experiment.

### Study protocol

**Figure 1.**
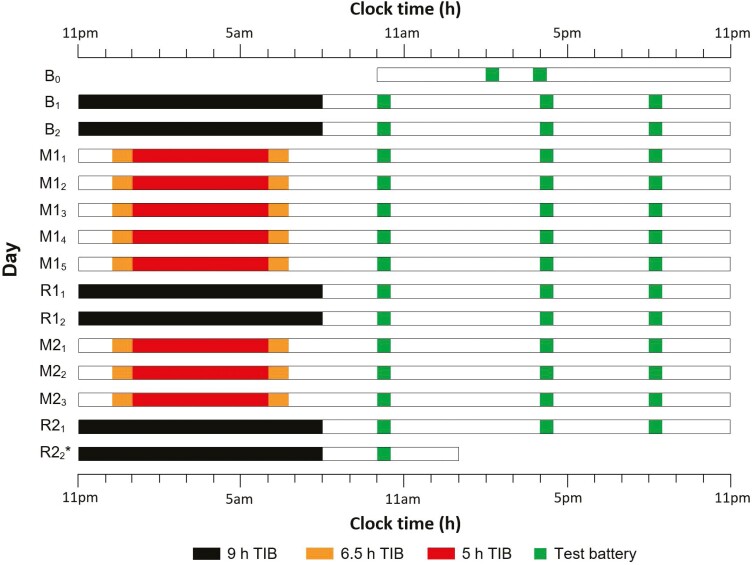
**Experimental protocol.** The study lasted for 15 days. All the participants started with two adaptation and baseline nights (B_1_ and B_2_) of 9-h TIB per night (black bars). This was followed by the first cycle of sleep manipulation for 5 nights (M1_1_ to M1_5_) and recovery sleep for 2 nights (R1_1_ and R1_2_; TIB = 9 h; black bars). The second cycle consisted of 3 nights of sleep manipulation (M2_1_ to M2_3_) and 2 nights of recovery sleep (R2_1_ and R2_2_; black bars). During both sleep manipulation periods, participants had nocturnal TIBs of 5 h (5 h group; red bars) or 6.5 h (6.5 h group; orange bars). A cognitive test battery (green bars) was administered daily at 10:00, 15:45–16:00, and 20:00, except during the first and last days of the protocols.

The NFS study series was aimed at characterizing adolescents’ neurobehavioral functions under different sleep schedules. In the current analysis, comparisons involved 2 groups of participants who underwent two periods of sleep restriction to 5 or 6.5 h that was below the minimum age-appropriate recommended duration of 8 h [29]. Specifically, the 15-day study (Figure 1), which was conducted in a boarding school during the year-end holiday period, started with two adaptation/ baseline nights of 9-h TIB (B_1_ and B_2_: 23:00–08:00), followed by two successive cycles of manipulation nights and recovery nights. The first cycle consisted of five nights of sleep opportunity manipulation (M1_1_-M1_5_: 01:00–06:00 for the 5-h group; 00:15–06:45 for the 6.5-h group) and ended with 2 nights of 9-h recovery sleep opportunity (R1_1_–R1_2_: 23:00–08:00), simulating a typical school week. The second cycle included three manipulation nights (M2_1_–M2_3_) and ended with two nights of recovery (R2_1_–R2_2_). Details on the boarding arrangement as well as the polysomnographic changes (Supplementary Table S1) from baseline to the sleep restriction and recovery nights are summarized in the Supplementary Materials. During the week prior to their stay at the boarding school, participants were required to follow a 9-h sleep schedule (23:00–08:00), and compliance was verified with actigraphy (Supplementary Table S1) [8, 9].

Sustained attention was assessed with a 10-min PVT [6] 2 times on the first day of the study (B_0_), and thereafter, 3 times daily (Figure 1). The PVT was the last task of a cognitive test battery that was administered in a classroom at 10:00, 15:45–16:00, and 20:00.

All the NFS study protocols were approved by the Institutional Review Board of the National University of Singapore and conducted according to the principles in the Declaration of Helsinki. Participants and their legal guardians provided written informed consent prior to their participation in the studies.

### Psychomotor vigilance task

During the 10-min PVT [6], a counter on the computer screen started counting at random intervals varying from 2000 ms to 10 000 ms. Participants were instructed to press the spacebar as quickly as possible whenever the counter started. A tone was presented via the participants’ earphones if they failed to respond 10 000 ms after stimulus onset. The primary outcome measure was the daily average of the number of lapses (response times ≥ 500 ms), as it is known to be one of the most sensitive measures to the effects of sleep loss [13, 30]. Secondary outcome measures of interest included the daily averages of median reaction time (RT) and standard deviation in reaction time (SD RT) in ms because these measures were also sensitive to the effects of partial sleep deprivation.

For each participant, linear regression was conducted to derive the slope of each PVT measure from the last baseline day (B_2_) to the last manipulation day in the first cycle (M1_5_) for quantifying the deterioration in vigilance during the first week of sleep restriction (i.e. slope_wk1_). Similarly, a slope (i.e. slope_wk2_) was derived for each participant for the second sleep restriction week using data from the last recovery day in the first cycle (R1_2_) to the last manipulation day in the second cycle (M2_3_). The difference between these two slopes (i.e. slope_wk2_–_wk1_) was used to indicate whether an individual’s vigilance deteriorated faster or slower during the second relative to the first week of sleep restriction, and in other words, was a measure of the vigilance vulnerability to recurrent cycles of sleep restriction.

PVT RT data for all three sessions on the last baseline day (B_2_) were fed into a mixed estimator function for generating DDM metrics. This function segments each decision-making process into the decision (cognitive) and non-decision (physiological response) components, quantifiable as the drift rate and non-decision time for the respective components (for a detailed conceptual and mathematical breakdown of this process, refer to [31]). This function collapses data from separate PVT sessions within the baseline day and generates the DDM metrics based on the day’s performance. This pipeline is a replica of the one-choice DDM paradigm used in Patanaik et al. [20, 22]. Of particular interest in this study are the DDM metrics generated for the last baseline day (B_2_), including the range in the non-decision time and the average in drift rate, which were used as variables to predict inter-individual differences in vigilance vulnerability during multi-night sleep restriction.

### Statistical analysis

Statistical analyses were conducted with SAS 9.4 (SAS Institute, Cary, NC). Group differences in the demographic and sleep-related measures taken at the screening stage (Table 1) were examined with independent-sample *t*-tests and chi-squared tests. Mixed models with week as a within-subject factor, group as a between-subject factor and slope as the dependent variable were used to test whether performance deterioration across the first and the second weeks of sleep restriction differed between the 5 h and the 6.5 h groups.

Multiple regression analyses were used to determine if PVT summary metrics (the number of lapses, median RT, or SD RT) and two DDM metrics (the average in drift rate and range in the non-decision time) derived from day B2 could predict inter-individual differences in vulnerability during 1 week of sleep restriction (slope_wk1_) and vulnerability to recurrent cycles of sleep restriction (slope_wk2_–_wk1_).

## Results

### Sample characteristics

The two groups did not differ significantly at the screening stage, except for actigraphically assessed TIB on weekdays and sleep efficiency (*p* = .02 and *p* < .001; Table 1). Nevertheless, the TIB difference was not found with the PSQI (*p* = .26), and importantly, no significant group difference was found for actigraphically assessed TST (*p* = .41).

### Vigilance deterioration during the first and second weeks of sleep restriction

A significant week × group interaction was found for all three PVT outcome variables (*p* < .007; Table 2). The two groups showed similar rates of increase in lapses, median RT, and SD RT in the first week of sleep restriction (*p* > .89), but during the second week, performance deteriorated faster in the 5 h group than the 6.5 h group (*p* < .001). Moreover, all three variables increased significantly faster in the second week of sleep restriction compared to the first week in the 5 h group (*p* < .001), but not in the 6.5 h group (*p* > .48).

**Table 2.** Deterioration in Psychomotor Vigilance Test (PVT) performance of the two groups in the first and second weeks of sleep restriction

	Slope_wk1_		Slope_wk2_		Week effect	Group effect	Week × group interaction
	Mean	SEM	Mean	SEM	*p*	*p*	*p*
Number of lapses							
5 h group	1.78	0.39	4.75	0.39	<.001	<.001	<.001
6.5 h group	1.77	0.39	1.90	0.39			
Median RT (ms)							
5 h group	11.75	21.54	123.73	21.54			
6.5 h group	15.84	21.17	5.70	21.17	.02	.009	.007
SD RT (ms)							
5 h group	63.38	26.28	303.40	26.28	<.001	<.001	<.001
6.5 h group	64.54	25.82	86.72	25.82			

RT = reaction time; SD RT = standard deviation in reaction time; mean and standard error of the mean (SEM) were derived from the mixed models.

### Predicting vigilance decrement during sleep restriction with baseline performance

**Figure 2.**
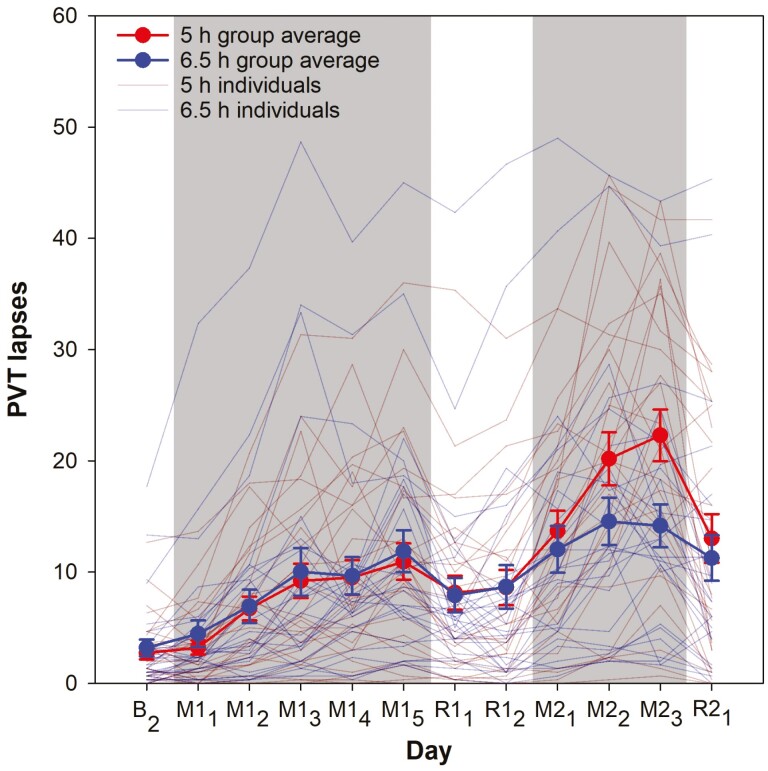
**Inter-individual differences in the number of Psychomotor Vigilance Test (PVT) lapses during two cycles of sleep restriction and recovery.** The averages of the 5 h and the 6.5 h groups are plotted in red and blue respectively. Individual data are shown in dark red for the 5 h group and dark blue for the 6.5 h group. Refer to Figure 1 legend for a detailed explanation of the study days.

**Figure 3.**
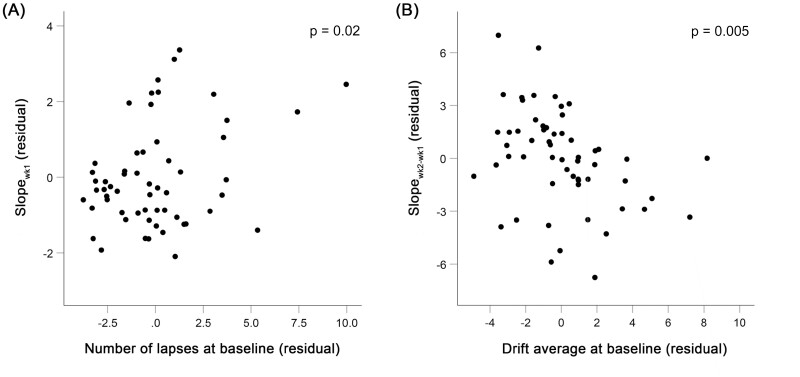
**Associations between baseline Psychomotor Vigilance Test (PVT) performance and deterioration in vigilance during the first and second weeks of sleep restriction.** (A) More lapses in attention at baseline were significantly associated with a faster increase in lapses during the first week of sleep restriction (*B* = 0.16, *SE* = 0.07, *p* = .02). This association was controlled for TIB and the two drift-diffusion modelings (DDM) parameters (non-decision time range and drift average) at baseline. (B) Higher drift average at baseline was significantly associated with a greater increment in the rate of lapse increase from the first to the second week of sleep restriction (*B* = −0.40, *SE* = 0.13, *p* = .005). This association was controlled for TIB, as well as the number of lapses and non-decision time range at baseline.

Prominent inter-individual differences in PVT performance were observed among adolescents during both the first and second cycles of sleep restriction (Figure 2). Faster accumulation of lapses during the first week of sleep restriction was significantly associated with more lapses at baseline (*p* = .02; Figure 3, A), but not the two baseline DDM metrics (*p* > .07; Table 3). None of the baseline PVT measures was found to be associated with the increase in median RT and SD RT across the first sleep restriction cycle (*p* > .16; Table 3).

**Table 3.** Predicting inter-individual differences in vigilance vulnerability to one and two weeks of sleep restriction with baseline Psychomotor Vigilance Test (PVT) metrics

	*B*	SE	*p*
Slope_wk1_			
Number of lapses			
Baseline number of lapses	0.16	0.07	**.02**
Baseline non-decision time range	25.48	13.92	.07
Baseline drift average	−0.03	0.07	.66
Median reaction time (RT)			
Baseline median RT	0.15	0.10	.16
Baseline non-decision time range	−20.65	173.31	.91
Baseline drift average	−0.70	0.95	.47
Standard deviation (SD) in RT			
Baseline SD RT	0.20	0.14	.17
Baseline non-decision time range	865.36	874.96	.33
Baseline drift average	0.54	3.82	.89
Slope_wk2–wk1_			
Number of lapses			
Baseline number of lapses	−0.45	0.13	**.001**
Baseline non-decision time range	−45.90	28.05	.11
Baseline drift average	−0.40	0.13	**.005**
Median RT			
Baseline median RT	−1.01	1.02	.33
Baseline non-decision time range	−9.76	1708.11	.99
Baseline drift average	−14.33	9.35	.13
SD RT			
Baseline SD RT	−0.48	0.27	.08
Baseline non-decision time range	−803.42	1685.52	.64
Baseline drift average	−20.61	7.37	**.007**

Slope_wk1_ quantified the changes in the number of lapses, median RT, and SD RT during the first week of sleep restriction. Slope_wk2–wk1_ quantified the difference in the rates of performance deterioration between the first and the second weeks of sleep restriction and thus, represented each participant’s vulnerability to two cycles of sleep restriction with intervening recovery sleep. Group was included as a covariate in all the regression models.

On the other hand, individuals with the faster accumulation of lapses in the second relative to the first week of sleep restriction were found to have lower drift and smaller number of lapses (*p* = .005 and .001; Table 3 and Figure 3, B) on the baseline day, while no significant association was found for non-decision time range at baseline (*p* = .11). Lower drift at baseline also predicted greater increment in the slope of SD RT from the first to the second week of sleep restriction (*p* = .007; Supplementary Figure S1), but the association was not significant for median RT (*p* = .13).

## Discussion

We used data from two multi-night sleep restriction protocols conducted on adolescents to compare the relative contributions of summary and DDM metrics at baseline in predicting vigilance declines. We found that declines in vigilance accrued more rapidly in the second cycle of partial sleep deprivation (after 2 nights of recovery sleep) than in the first cycle, particularly when sleep restriction was severe. Furthermore, average drift predicted faster declines in vigilance performance from the first to the second week on two out of three measures of interest (lapses and SD RT), but not the decrement in the first week. The reverse was true of lapses at baseline, suggesting that summary metrics and DDM measures predict different facets of vulnerability to sleep curtailment.

Several published studies have shown that baseline PVT lapses are the most consistent predictors of vulnerability to total and partial sleep loss. Chua et al. [19] exposed a group of healthy volunteers to 26 h of total sleep deprivation, and demonstrated that vulnerable (bottom tertile) performers differed significantly from resilient (top tertile) performers in the number of lapses, average RT, and SD RT in baseline PVT performance. They concluded that sleep deprivation amplifies subtle individual differences in vigilance that are already present in the baseline, well-rested state. In a later study using linear discriminant modeling, Chua et al. [24] were able to classify participants into performance bands with relatively high sensitivity (78%) and specificity (86%) based on a reliable set of baseline PVT features. Others have also reported that pattern recognition using PVT and other data from a single testing session at baseline could be used to classify performance impairment after a night of total sleep deprivation [32]. Regarding vulnerability during multi-night partial sleep loss, in a large (*N* = 306) sample of healthy adults undergoing sleep restriction to 4 h per night for 5 nights, Galli et al. [17] reported that lapses in vigilant attention at baseline were the strongest predictor of subsequent decrements.

Other than summary task metrics, some variables derived from DDM appear useful in predicting vigilance vulnerability during total sleep loss. Patanaik et al. [22] used summary PVT metrics, DDM parameters, and wavelet metrics from 180 participants who had undergone a night of total sleep deprivation to classify vulnerable and resilient individuals. They found that the DDM metrics of diffusion drift, and non-decision time range emerged among the top predictive features. Studies have shown that drift rates in general [33, 34] and at baseline [20, 22] are correlated to PVT performance during sleep deprivation, demonstrating the high consistency and reliability of this association. The present study is in agreement with Patanaik et al. [20, 22], showing that drift rate at baseline is indicative of how resilient an adolescent is to performance deficit during recurrent sleep restriction, albeit non-decision time did not have any predictive value in the current analysis.

Taken together, our results support and extend the existing literature by demonstrating the robustness of using summary PVT and DDM metrics to predict vigilance vulnerability among adolescents undergoing multi-night sleep restriction. Interestingly, the predictive value of baseline lapses was only seen for the first week of sleep restriction, whereas DDM metrics were associated with the additional decrement in the second cycle. These results suggest that there may be mechanistic differences between short- and longer-term vulnerability, which is further supported by the significant difference in the slope of performance declines between weeks 1 and 2 of the protocol for the 5 h group. Recurrent cycles of sleep restriction in adolescents may thus expose instability that is more specifically related to information accumulation, possibly as the 2 nights of recovery sleep may have lowered the propensity to lapse in certain individuals.

Here, we showed for the first time that baseline data contain more predictive information (beyond lapses, in the form of DDM metrics) that capture vulnerability on different time scales—that the predictive strength of lapses and DDM metrics on subsequent PVT performance after sleep deprivation is contingent on the length of the sleep restriction period. As this is the first time such an effect has been reported, further replication is needed to confirm this effect and explore its underlying mechanisms.

Over the last century, there has been a steady trend towards adolescents getting less sleep [35], although lockdowns necessitated by the COVID-19 pandemic may have recently reversed this decline by increasing sleep opportunities [36, 37]. In Asia, sleep curtailment is common, with adolescents reporting shorter sleep on school nights and greater daytime sleepiness than their counterparts in Europe and North America [38]. For example, 85% of adolescent students in Singapore report sleeping less than the recommended 8–10 h a night [29] on school nights [39]. While the problem of sleep curtailment in adolescents arises from a complex interplay of biological and psychosocial factors [40], prioritization of school work and academic achievement is a key factor for sacrificing TIB in some Asian societies [41]. Paradoxically, because of its effects on vigilance and cognition, insufficient sleep itself negatively affects school performance [42, 43], rendering this a questionable sacrifice at best.

Against this backdrop, the ability to predict vulnerability to sleep loss may be a useful aid to those balancing life commitments against sleep needs. For the especially vulnerable, understanding this trait may aid them in making more informed decisions about their time use, and how much time to spend in bed.

### Limitations and future studies

The current sample consisted of adolescents. Thus, whether in other age groups, baseline summary PVT and DDM metrics differentially predict inter-individual differences in vigilance vulnerability during a single week or more weeks of sleep restriction remains to be addressed in future studies. Moreover, given that vigilance is the cognitive domain most impaired by sleep loss [1, 7, 13, 44], most published studies and the current investigation focused on finding predictors of vigilance performance during total and partial sleep deprivation. More work is required to investigate whether, for other cognitive domains, performance at a well-rested state can predict performance in the same task in a sleep-restricted or sleep-deprived state. Finally, as the analyses performed here in this paper were post hoc, they should be treated as exploratory, and confirmatory investigation is needed.

## Conclusions

In a similar fashion to adults, summary and DDM metrics at baseline may be useful predictors of vulnerability to sleep restriction in adolescents, indicating that these individual differences are relevant to performance even at that maturational stage.

## Supplementary Material

zpac040_suppl_Supplementary_Materials

## Data Availability

The data that support the findings of this study are not openly available and is presently stored in the data servers of the Centre for Sleep and Cognition. However, data are available from the first author upon reasonable request from other researchers that are able to provide a clear study hypothesis.

## Footnotes

1 The NFS studies aimed at characterizing neurobehavioral changes during different sleep schedules among adolescents. These quasi-experiments were conducted at a boarding school, and participants’ sleep and testing schedules were carefully monitored by the research staff.

## Notes

This paper is part of the David F. Dinges Festschrift Collection. This collection is sponsored by Pulsar Informatics and the Department of Psychiatry in the Perelman School of Medicine at the University of Pennsylvania.
