# Supplementary material for: Predicting vigilance vulnerability during 1 and 2 weeks of sleep restriction with baseline performance metrics
Source: Sleep Adv. 2022 Oct 25;3(1):zpac040. doi: 10.1093/sleepadvances/zpac040 (PMC10104386; doi:10.1093/sleepadvances/zpac040)

**Predicting vigilance vulnerability during one and two weeks of sleep restriction with baseline performance metrics**

June C. Lo^1,2^; Jit Wei A. Ang^1^; Tiffany B. Koa^1^; Ju Lynn Ong^1^; Julian Lim^1,2^

^1^ Centre for Sleep and Cognition, Yong Loo Lin School of Medicine, National University of Singapore, Singapore

^2^ Department of Psychology, National University of Singapore, Singapore

Corresponding authors:

Dr. June Chi-Yan Lo

Centre for Sleep and Cognition

Yong Loo Lin School of Medicine,

National University of Singapore,

MD1 Tahir Foundation Building, 12 Science Drive 2,

Singapore 117549

Phone: (+65) 66016146

E-mail: june.lo@nus.edu.sg

Dr. Julian Lim

Centre for Sleep and Cognition

Yong Loo Lin School of Medicine,

National University of Singapore,

MD1 Tahir Foundation Building, 12 Science Drive 2,

Singapore 117549

Phone: (+65) 66011956

E-mail: julian.lim@nus.edu.sg

**Supplementary Materials**

**Pre-experiment sleep schedule at home**

In the week preceding the experimental phase, participants were instructed to go to bed at 23:00 and wake up the following day at 08:00 for circadian entrainment and to minimise potential effects of prior sleep restriction on cognitive performance during the experiment. Compliance was verified with actigraphy (Table S1).

**Table S1 Sleep timing and durations one week prior to the experiment**

|  | **5 h group** | | **6.5 h group** | |
| --- | --- | --- | --- | --- |
|  | **Mean** | **SD** | **Mean** | **SD** |
| Bedtime (clock time) | 23:00 | 00:07 | 23:05 | 00:21 |
| Wake time (clock time) | 08:03 | 00:21 | 08:05 | 00:27 |
| Time in bed (h) | 9.04 | 0.28 | 8.99 | 0.31 |
| Total sleep time (h) | 7.92 | 0.45 | 7.37 | 0.42 |

**Experimental phase of the study**

Participants resided in a boarding school, and males and females were housed in separate blocks. All rooms were twin shares, air-conditioned, and came with ensuite bathroom. Blackout panels were fitted over the bedroom windows to ensure that sleep was not disrupted by external light. Apart from scheduled times for sleep, meals, showers and cognitive testing, participants were free to spend their time as they wished, such as reading, watching videos, playing cards, in a common room that received natural as well as artificial lighting. Participants were not allowed to leave the boarding school during the experiment and were constantly monitored by research staff during the wake periods. They were not allowed to engage in strenuous physical activity, consume caffeine products, and take naps.

**Figure S1 Association between lower drift average at baseline and greater increment in response speed variability (SD RT) from the first to the second week of sleep restriction.** The association (*B* = -20.61, *SE* = 7.37, *p* = 0.007) was controlled for TIB, as well as SD RT and non-decision making time range at baseline.


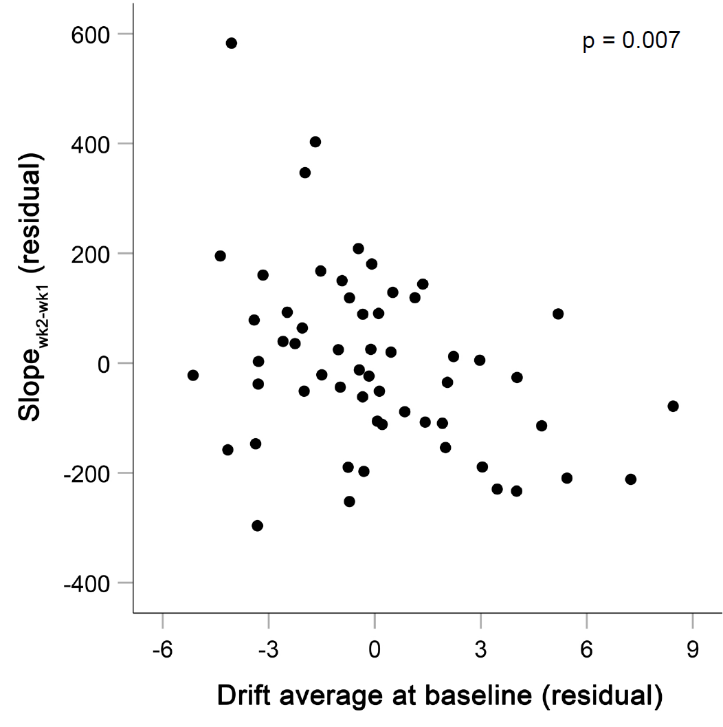

Supplement: zpac040_suppl_Supplementary_Materials [file zpac040_suppl_supplementary_materials.docx]
